# An experimental examination of catastrophizing-related interpretation bias for ambiguous facial expressions of pain using an incidental learning task

**DOI:** 10.3389/fpsyg.2014.01002

**Published:** 2014-09-17

**Authors:** Ali Khatibi, Martien G. S. Schrooten, Linda M. G. Vancleef, Johan W. S. Vlaeyen

**Affiliations:** ^1^Research Group on Health PsychologyKU Leuven, Leuven, Belgium; ^2^Center for Health and Medical Psychology, Örebro UniversityÖrebro, Sweden; ^3^Department of Clinical Psychological Science, Maastricht UniversityMaastricht, Netherlands

**Keywords:** painful facial expressions, interpretation bias, indirect measures, incidental learning task, direct measures, pain catastrophizing

## Abstract

Individuals with pain-related concerns are likely to interpret ambiguous pain-related information in a threatening manner. It is unknown whether this interpretation bias also occurs for ambiguous pain-related facial expressions. This study examined whether individuals who habitually attach a catastrophic meaning to pain are characterized by negative interpretation bias for ambiguous pain-related facial expressions. Sixty-four female undergraduates completed an incidental learning task during which pictures of faces were presented, each followed by a visual target at one of two locations. Participants indicated target location by pressing one of two response keys. During the learning phase, happy and painful facial expressions predicted target location. During two test phases, morphed facial expressions of pain and happiness were added, equally often followed by a target at either location. Faster responses following morphs to targets at the location predicted by painful expressions compared to targets at the location predicted by happy expressions were taken to reflect pain-related interpretation bias. During one test phase, faces were preceded by either a safe or threatening context cue. High, but not low, pain-catastrophizers responded faster following morphs to targets at the location predicted by painful expressions than to targets at the other location (when participants were aware of the contingency between expression type and target location). When context cues were presented, there was no indication of interpretation bias. Participants were also asked to directly classify the facial expressions that were presented during the incidental learning task. Participants classified morphs more often as happy than as painful, independent of their level of pain catastrophizing. This observation is discussed in terms of differences between indirect and direct measures of interpretation bias.

## Introduction

Pain-related behaviors, such as facial expressions, provide information about one's current feelings and situation to others (Williams, 2002). However, pain behavior can be ambiguous, not always providing a clear signal of pain or somatic threat (Pincus and Morley, 2001). Interpreting ambiguous pain signals in a threatening manner might be adaptive, as it reflects early threat detection and facilitates fast action when needed (Ohman and Mineka, 2001). However, in some conditions, such negative interpretation bias might lose its functional value (Vancleef et al., 2009). Especially relevant to pain and maladaptive pain responding is whether negative interpretation bias of ambiguous pain behavior depends on the meaning attached to pain. It has been suggested that individuals who habitually attach a catastrophic meaning to pain perceive others' pain as more intense, and feel more distress when observing others in pain than individuals who catastrophize less about pain (Sullivan et al., 2006; Goubert et al., 2011). Biased interpretation of ambiguous pain-related information, such as words related to pain and somatic threat, has found to be associated with individuals' levels of pain-related anxiety, pain catastrophizing, and pain-related fear in healthy individuals (Pincus and Morley, 2001; Keogh and Cochrane, 2002; McKellar et al., 2003; Vancleef et al., 2009). In the current study, we investigated biased interpretation of ambiguous pain-related facial expressions (i.e., morphed facial expressions of pain and happiness) in healthy volunteers, taking individual differences in level of pain catastrophizing into account.

Besides the observer's level of pain catastrophizing, interpretation bias regarding others' pain behavior might also depend on available context information. It has been shown that the processing of facial expressions is influenced by emotional context information (De Gelder et al., 2006). Furthermore, healthy individuals' tendency to classify ambiguous pain-related facial expressions as painful has shown to be especially enhanced when these expressions are preceded by negative priming words (Yamada and Decety, 2009). Therefore, a second aim of the current study was to examine the influence of physically threatening contextual information on interpretation bias for ambiguous facial expressions.

Direct measures of interpretation bias, such as direct classification tasks, have frequently been used in the study of cognitive biases related to pain and threat (e.g., Richards et al., 2002; Liossi et al., 2012), but also have been criticized. One of the problems with the direct measures is their susceptibility to self-presentation biases (Nisbett and Wilson, 1977; Hirsch and Mathews, 1997). Indirect measures of interpretation bias avoid this problem by inferring interpretations from behavioral response patterns. Therefore, we applied an indirect task, and more specifically an incidental learning paradigm (cf. Yoon and Zinbarg, 2008) in addition to a direct classification task, to examine interpretation bias for pain-related ambiguous facial expressions. This is the first published study that uses the incidental learning task to examine pain-related interpretation bias.

In sum, we hypothesized that healthy individuals, and especially high pain catastrophizers, interpret morphed facial expressions of pain and happiness in a negative, pain-related manner. We further hypothesized that this bias will be enhanced when morphs are presented in a threatening context.

## Methods

### Participants

Sixty-four Dutch-speaking female undergraduates from the University of Leuven took part in this study. Exclusion criteria were history of chronic pain, presence of acute pain, and uncorrected visual problems. Three participants were excluded from further analyzes because their dataset was incomplete due to technical problems. The final sample consisted of 61 participants (mean age = 18.37 years, *SD* = 0.7).

Groups representing high (*n* = 29) and low (*n* = 32) pain catastrophizers were formed based on the final sample's median score (17) on the Pain Catastrophizing Scale (see Sections Pain Catastrophizing Scale and Apparatus). The high pain catastrophizers' mean PCS score (25.9; *SD* = 6.3) was in the 9th decile of norm scores for female, Belgian, Dutch-speaking undergraduate students; the low catastrophizers' mean PCS score (11.4; *SD* = 5.0) was in the 3rd decile of these norm scores (Van Damme et al., 2000).

The experiment was approved by the ethical committee of the faculty of psychology, University of Leuven, Belgium. All participants took part based on informed consent, in exchange for a course credit or money (7€).

### Pain catastrophizing scale

Participants completed the Dutch version of the Pain Catastrophizing Scale (PCS; Sullivan et al., 1995; Van Damme et al., 2000). The PCS consists of 13 items describing different thoughts and feelings that may be associated with pain. Participants indicate the degree to which they have each of those feelings or thoughts on a 5-point Likert scale (0 = not at all; 4 = all the time). We calculated a total PCS score with a range of 0-52 by summing the 13 item scores. Higher total scores reflect higher levels of pain catastrophizing. In our final sample PCS total scores ranged between 0-42 (mean = 18.1, *SD* = 6.3). The psychometric properties of the Dutch version of the PCS have been approved for different populations (reported Cronbach's Alpha in Dutch-speaking population >0.85, Van Damme et al., 2000).

### Stimulus materials

#### Pictorial face stimuli

Pictorial face stimuli were presented during the incidental learning task (see Section Incidental Learning Task) and the direct classification task (see Section Direct Classification Task). Colored photographs (height 6 cm × width 4.5 cm) of happy and painful facial expressions from 54 actors (30 male; young Caucasian adults and racially congruent to the participants) were obtained from two databases (Roy et al., 2007; Langner et al., 2010). On all photographs, head and eye-gaze were directed forward and the head filled most of the picture. All images had the same size and the relative size of head was the same for all images. Non-facial features were removed and replaced with a uniform gray background, because this information might distract from expression processing (Nusseck et al., 2008).

A pilot study with 20 female undergraduates (mean PCS = 17.7, *SD* = 5.9; mean age = 18.4, *SD* = 0.6) from the same population as the experimental sample (but who did not take part in the actual experiment) was conducted to select the face stimuli. During this pilot study, participants rated 180 face stimuli on four different scales (Simon et al., 2008): the intensity of *happiness* in the expression on a 6-point Likert scale (0 = not happy at all; 5 = extremely happy), the intensity of *pain* in the expression on a 6-point Likert scale (0 = not painful at all; 5 = extremely painful), the extent of *pleasantness* of the expression on a 9-point Likert scale (−4 = extremely unpleasant; 4 = extremely pleasant), and the extent of *arousal* of the expression on a 9-point Likert scale (−4 = completely calm; 4 = extremely aroused). Based on these ratings (data provided in Table S1, online only), 16 painful and 16 happy expressions from 32 actors (16 females; eight male and eight female actors expressed pain; the other half of the actors expressed happiness) were selected to be presented as prototype (unmorphed) expressions during the actual experiment. Sixteen morphed expressions were created by morphing the pictures of 16 painful and 16 happy expressions from 16 other actors (all white Caucasians; eight females), using Fanta-Morph software (Delux, 3.4.2^1^). More specifically, for each actor, a painful expression was paired with a happy expression. For each of the resulting 16 pairs, the software produced 60 frames (transition from painful to happy expression) from which five different frames were selected, each consisting of a similar amount (percentage) of painful and happy expression. In the process of creating and selecting the morphs 10 experts in the coding of facial expressions (FACS coding) were asked for their independent opinion and expert view. They were asked to select for each of the 16 pairs the most ambiguous morph out of the five created morphs and to rate its perceptual quality on a 5-point Likert scale (0 = Very poor, 4 = Very good). The morphs selected by at least half of the experts, as being the most ambiguous morph for that specific pair, and with sufficient quality (mean rating = 3.66, *SD* = 0.35) were selected for the present study. Examples of the selected stimulus materials are presented as Supplementary Materials (Figure S1; online only).

Finally, all participants of the actual experiment rated at the end of the experimental lab session the ambiguity of all facial stimuli that were presented during the interpretation bias tasks (see Section Procedure) on a 100 mm VAS (1 = “minimum level of ambiguity” anchored on the left, 10 = “maximum level of ambiguity” anchored on the right). Morphs were rated as more ambiguous (mean = 6.15, *SD* = 1.9) than happy expressions [mean = 1.34, *SD* = 0.6, *t*_(60)_ = 20.55, *p* < 0.001] and painful expressions [mean = 1.57, *SD* = 0.7, *t*_(60)_ = 18.86, *p* < 0.001]. There was no significant difference between high and low pain catastrophizers' rating of ambiguity in morphed expressions [High PCS: mean = 6.18, *SD* = 1.9; Low PCS: mean = 6.11, *SD* = 1.8; *t*_(59)_ = 0.13, *p* = 0.9]; happy expressions [High PCS: mean = 1.43, *SD* = 0.7; Low PCS: mean = 1.26, *SD* = 0.4; *t*_(59)_ = 1.18, *p* = 0.2] and painful expressions [High PCS: mean = 1.46, *SD* = 0.7; Low PCS: mean = 1.66, *SD* = 0.8; *t*_(59)_ = 1.05, *p* = 0.3].

#### Context cues

Context cues were presented during the incidental learning task (see Section Incidental Learning Task). Context cues were 16 colored photographs (height 4 cm × width 6 cm) of which eight of them depict a hand in a physically threatening situation, and eight a hand in a nonthreatening situation as obtained from a database developed by Jackson et al. (2005). Threatening and non-threatening context cues were matched in terms of positioning and background. This selection was based on threat ratings on a 10-point Likert scale as provided with the original database (by Jackson et al., 2005). For the threat-related photos, threat ratings were between 5.6 and 7.5 (mean = 6.22, *SD* = 0.67) and for non-threatening photos less than 0.18 (mean = 0.06, *SD* = 0.04). Examples of contextual cues are presented as Supplementary Materials (Figure S2; online only 2).

### Interpretation bias tasks

#### Incidental learning task

**Figure 1:**
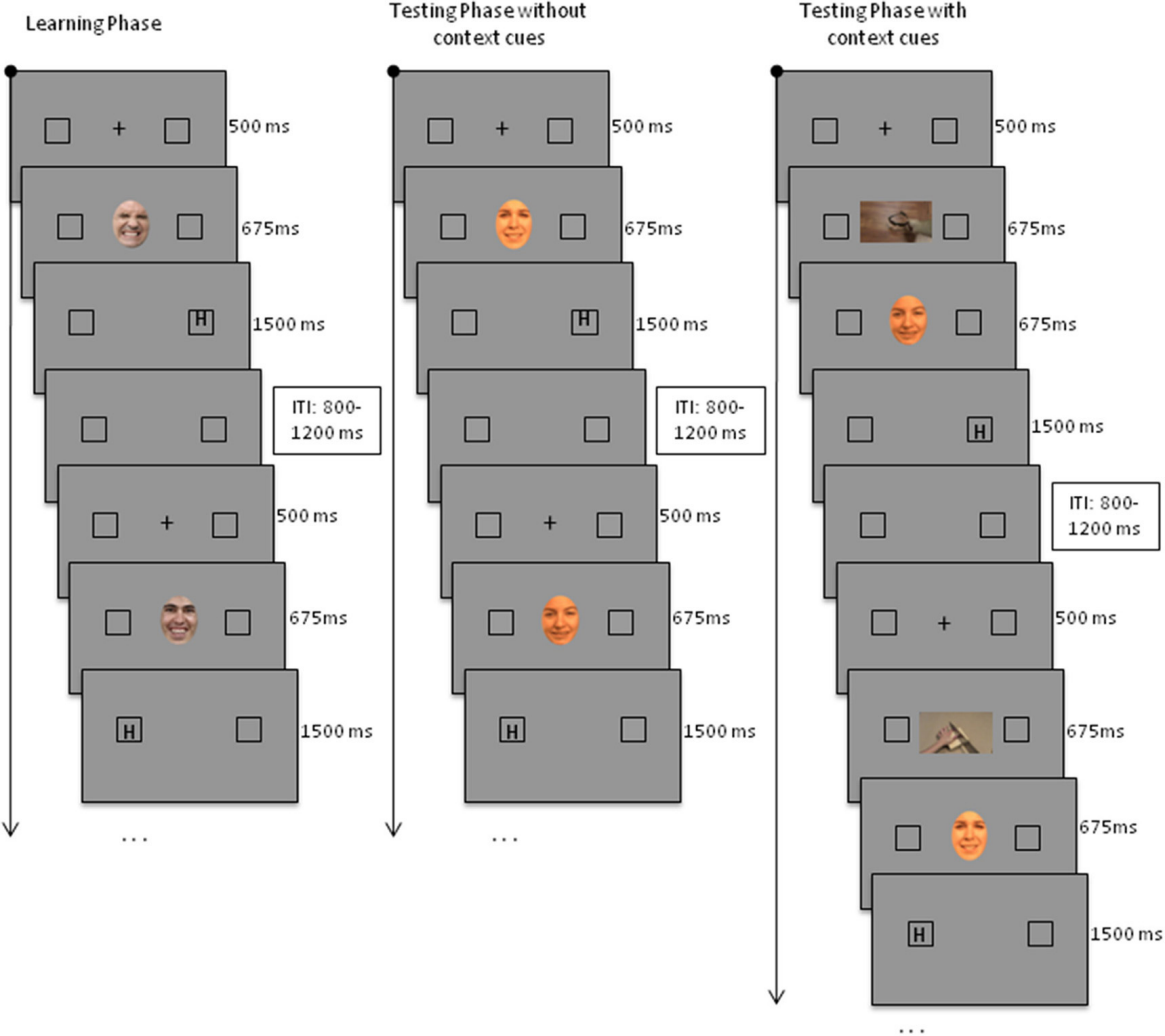
**Typical trial configuration for the learning and testing phases of the incidental learning task**.

***General***. The incidental learning task (cf. Yoon and Zinbarg, 2008) consisted of three phases: a learning phase and two testing phases (Figure 1). During the learning phase, unmorphed painful and happy expressions were presented one by one, followed by a target at one of two predefined locations. Expression type predicted the target's location and participants were expected to learn this association. During the testing phases, morphed facial expressions were presented in addition the unambiguous happy and painful expressions. The rationale behind the incidental learning task is that following the presentation of a morphed facial expression, participants respond faster to targets at the location predicted by painful expressions if they interpreted the expression as painful. On the other hand, they are expected to respond faster to targets at the location predicted by happy expressions if they interpreted the morphed expression as happy. So, faster reactions following morphed expressions to targets at the location predicted by painful expressions in comparison with the location predicted by happy expressions were taken as indicative of pain-directed interpretation of morphed facial expressions.

***Learning phase (Figure 1; left panel)***. During the learning phase, each trial started with a black central fixation cross on a gray background and two square position markers (black frames, 1 × 1 cm), one at the left and one at the right of the fixation cross. The inner edge of the target position-marker distanced 12 cm (horizontal axis) from the fixation cross. The fixation cross was presented for 500 ms and then replaced by an unambiguous happy or painful facial expression. This expression was presented for 675 ms and was immediately followed by a target letter “H” (0.85 × 0.85 cm). For half of the participants, (1) happy expressions were followed by a target at the left side of the fixation cross in 80% of the trials (i.e., *location predicted by happy expressions*) and at the right side in 20% of the trials (i.e., *location predicted by painful expressions*) and (2) painful expressions were followed by a target at the right of the fixation cross in 80% of the trials (i.e., *location predicted by painful expressions*) and at the left side in 20% of the trials (i.e., *location predicted by happy expressions*). For the other participants, right target location was predicted by happy expressions and left target location by painful expressions. Participants' task was to indicate on each trial the target's position as quickly and accurately as possible, by pressing the corresponding key on the response box (i.e., left key to left target; right key to right target). So, for half of the participants the left key was associated with responses to targets at the location predicted by happy expressions and the right key with responses to targets at the location predicted by painful expressions; for the other participants this mapping was reversed. As soon as a response was given, or after 3000 ms, the screen was refreshed and the next trial was started. The learning phase consisted of two blocks, each consisting of 32 trials (16 happy and 16 painful expressions). Each individual expression was presented twice, once during each trial block. Trials were presented in a different random order for each participant.

***Test phase without context cues (Figure 1; middle panel)***. The test phase without context cues was similar to the learning phase, except that 16 morphed expressions were presented, equally often followed by a target at the left or the right side of the screen—together with eight happy expressions, always followed by a target a the location predicted by happy expressions during the learning phase, and with eight painful expressions, always followed by a target a the location predicted by painful expressions during the learning phase. These painful and happy expressions were randomly chosen from the 32 expressions that were presented during the learning phase and were the same for all participants. The trials with painful and happy expressions served as additional learning/retention trials. The test phase without context cues consisted of one block with 32 trials (16 morphs, eight happy, eight painful). Each individual expression was presented once. Trials were presented in a different random order for each participant.

***Test phase with context cues (Figure 1; right panel)***. The test phase with context cues only differs from the one without context cues in that after 500 ms, the fixation cross was first replaced by a context cue (i.e., picture of hand in either a threatening or nonthreatening situation). After 675 ms, the context cue was replaced by the facial expression. The rest of the trial was the same as for trials during the test phase without context cues. The test phase with context cues consisted of two blocks, each consisting of 32 trials (16 morphs, eight happy, eight painful). Each individual expression was presented twice, once preceded by a threatening cue and once by a non-threatening cue. Trials were presented in a different random order for each participant.

#### Direct classification task

Each trial of the direct classification task (Liossi et al., 2012) started with a fixation cross at the center of the computer screen. The cross was presented for 500 ms and then replaced by a happy, painful, or morphed facial expression. Each facial picture was presented for 675 ms and then replaced by two Dutch words, one besides the other, representing the two choice alternatives (“Painful” and “Happy”). All 32 photos that were presented during the testing phases of the incidental learning task were presented once, in a different random order for each participant. Participants' task was to indicate whether the facial expression was a happy or a painful one, by pressing the spatially corresponding response key on the response box. The position of the choice alternatives on the screen, and so the assignment of the response keys, was counterbalanced between participants. A higher number of morphs classified as painful than as happy is considered to reflect a negative interpretation bias.

### Apparatus

Task presentations, and logging of button presses were controlled by a Dell Optiplex 755 computer (OS: windows XP; 2 GB RAM; Intel Core2 Duo processor at 2.33 GHz; ATI Radeon 2400 graphics card with 256 MB of video RAM), running Affect 4.0 software (Spruyt et al., 2010) and connected to a 19” CRT DELL monitor (75 Hz vertical refresh rate; refresh duration: 13.3 ms/frame, image resolution 1280 × 1024), and a two button response box (via parallel port).

### Procedure

Participants were individually tested in a dimly lit testing room. They were informed that the experiment targeted the relationship between concentration and performance and signed the informed consent form. They were seated in front of the computer screen (viewing distance ≈ 60 cm). So the visual angle of the facial expressions to be presented on the computer screen was ~5.7° (vertically) and ~4.3° (horizontally), and of the context cues ~3.8° (vertically) and ~5.7° (horizontally). Participants positioned their hands on the response box, with their right index finger on the right response key and the left index finger on the left response key.

Then instructions for the incidental learning task were given. Participants were not informed about the to-be-learned associations. They were informed that the task would be followed by questions regarding the faces presented during the experiment. If all instructions were clear, the incidental learning task was started, with the learning phase followed by the two test phases (one without and one with context cues). The order of the test phases was counterbalanced between subjects. After each block of trials there was a short break during which participants were given the opportunity to relax and close their eyes for a minute.

After completion of the incidental learning task, the following questions were presented one by one: (1) *What different facial expressions did you see?* (2) *During the previous task you saw pictures of hands in different situations. Those situations can be divided into two or more general categories. To what different categories did the observed situations belong?* (3) *When a HAPPY face was presented, did the letter “H” more often appear on the right, more often on the left, or as often on either location?* and (4) *When a PAINFUL face was presented, did the letter “H” more often appear on the right, more often on the left, or as often on either location?* Whether participants were aware of the to-be-learned contingency between cue type (expression) and target location was derived from their answers to the third and fourth question.

After this assessment, all participants performed the direct classification task. Since performing the direct classification task could influence learning during the incidental learning task, the order of incidental learning task and direct classification task was not counterbalanced.

Finally, participants were asked to rate the ambiguity of the facial stimuli used in both interpretation bias tasks. See also Section Pictorial Face Stimuli.

Two days after the lab session, participants were invited by Email to complete as soon as possible but within 2 days via a secure online survey system a battery of questionnaires (EFS online survey), including demographical questions (e.g., age) and the Dutch version of the PCS. As soon as they had completed the questionnaires, participants received their compensations. When the data of all participants were collected, participants were informed about the experimental details and the aims of the study.

## Results

### Incidental learning task

#### Data preparation

Trials with incorrect responses were excluded from final analyzes. Trials with correct responses deviating more than 2.5 SDs from the individual's mean correct RT (per phase) were considered RT outliers and were also excluded. Percentages of excluded responses (% incorrect responses based on all responses; % RT outliers based on all correct responses) are reported at the beginning of each section, for each phase separately. The reported analyzes are on mean correct RTs after exclusion of outlier responses.

#### Learning phase

During the learning phase, 4.2% of the responses were excluded (1.7% incorrect responses; 2.5% RT outliers). Mean RTs (Table 1, top rows) were subjected to an ANOVA with expression type (2: painful vs. happy) and target location (2: location predicted by painful expressions vs. location predicted by happy expressions) as within-subjects factors and PCS group (2: high vs. low) as between-subjects factor. As expected, there was a significant expression type × target location interaction, *F*_(1, 59)_ = 18.0, *p* < 0.001, η^2^_*p*_ = 0.23, suggesting that participants learned the association between expression type and target location. Following painful expressions, RTs were significantly faster to targets at the location predicted by painful expressions than to targets at the location predicted by happy expressions, *t*_(60)_ = 4.1, *p* < 0.001, *Cohen's d* = 0.8. Following happy expressions, RTs were somewhat faster to targets at the location predicted by happy expressions than to targets at the location predicted by painful expressions, though non-significantly so, *t*_(60)_ = 1.0, *p* = 0.3, *Cohen's d* = 0.01. There was no other significant effect, indicating that the learning effect did not depend on participants' level of catastrophizing.

**Table 1:** **Mean reaction times (ms; mean ± s.e.m.)^a^ for each phase of the incidental learning task, separately for those scoring low and high on the Pain Catastrophizing Scale (PCS) and separately for those who were aware and unaware about the to-be learned contingency between expression and target location**.

**Phase**	**Context cue**	**Expression**	**Target location**	**Groups**			
				**Contingency aware**		**Contingency unaware**	
				**Low PCS**	**High PCS**	**Low PCS**	**High PCS**
				***n* = 24**	***n* =19**	***n* = 8**	***n* = 10**
Learning	n/a	Painful	Location predicted by painful faces, not by happy faces	345.6 ± 12.3	353.8 ± 13.9	315.3 ± 21.4	349.7 ± 19.1
			Location predicted by happy faces, not by painful faces	364.4 ± 14.4	368.8 ± 16.2	370.6 ± 24.9	392.0 ± 22.3
	n/a	Happy	Location predicted by painful faces, not by happy faces	364.0 ± 14.1	373.2 ± 15.8	316.6 ± 24.4	344.5 ± 21.8
			Location predicted by happy faces, not by painful faces	351.5 ± 12.6	353.8 ± 14.2	323.1 ± 21.9	365.7 ± 19.6
Test without Context Cues	n/a	Morph	Location predicted by painful faces, not by happy faces	329.4 ± 8.7	320.1 ± 12.9	293.9 ± 17.3	330.9 ± 15.4
			Location predicted by happy faces, not by painful faces	322.1 ± 15.1	347.9 ± 17.0	306.8 ± 18.6	323.8 ± 16.7
Test with Context Cues	Non-threatening cues	Morph	Location predicted by painful faces, not by happy faces	343.7 ± 11.2	340.9 ± 12.5	328.0 ± 17.9	357.6 ± 16.0
	Threatening cues		Location predicted by painful faces, not by happy faces	344.6 ± 12.1	335.1 ± 13.6	324.2 ± 18.7	347.9 ± 16.7
			Location predicted by happy faces, not by painful faces	341.7 ± 12.6	330.2 ± 14.2	317.3 ± 14.9	348.2 ± 13.3

a *Only correct RTs after exclusion of outlier responses were included*.

Since a number of learning theorists emphasize the importance of contingency awareness (e.g., Mitchell et al., 2009), we decided to include contingency awareness as a between subjects factor in the analyzes. This enables us to test whether learning the association between target location and type of facial expressions is influenced by the awareness of the contingencies between both stimuli. Forty-three participants of the final sample answered both the third and fourth awareness check question (see Section Procedure) correctly and were categorized as contingency aware (24 low-PCS; 19 high-PCS). The other participants answered both questions incorrectly and were categorized as contingency-unaware (8 low-PCS; 10 high-PCS)^2^. Adding awareness (2: cue-target contingency aware vs. unaware) as a between-subjects factor to the ANOVA with expression type, target location, and PCS group as factors revealed a significant interaction between expression type and target location, *F*_(1, 59)_ = 15.6, *p* < 0.001, η^2^_*p*_ = 0.21, that was no further modified by level of pain catastrophizing and/or awareness. Although the three-way interaction between expression type, target location, and awareness did not reach significance, *F*_(1, 57)_ < 0.02, *p* = 0.96, η^2^_*p*_ < 0.001, the interactions between awareness and expression type and between awareness and target location did, *F*_(1, 57)_ = 5.0, *p* = 0.02, η^2^_*p*_ = 0.08 and *F*_(1, 57)_ = 8.3, *p* = 0.005, η^2^_*p*_ = 0.13, respectively.

Therefore, we conducted the above mentioned ANOVA per contingency-awareness group. Contingency-*aware* participants showed the expected interaction between expression type and target location *F*_(1, 42)_ = 13.5, *p* = 0.001, η^2^_*p*_ = 0.24, indicating that they learned the association between expression type and target location. Following painful expressions, they were significantly faster to targets at the location predicted by painful expressions than the other location *t*_(42)_ = 2.6, *p* = 0.01, *Cohen's d* = 0.4. Following happy expressions, they were significantly faster to targets at the location predicted by happy expressions than the other location, *t*_(42)_ = 2.0, *p* = 0.05, *Cohen's d* = 0.3. There was no other significant interaction or main effect *Fs* < 1, *ps* > 0.5.

Contingency *unaware* participants also showed an interaction between expression type and target location, *F*_(1, 17)_ = 4.8, *p* < 0.04, η^2^_*p*_ = 0.22 [superseding a main effect of target location, *F*_(1,17)_ = 12.6, *p* = 0.002, η^2^_*p*_ = 0.43]. Following painful expressions, they were faster to targets at the location predicted by painful expressions than the other location, *t*_(17)_ = 3.48, *p* = 0.003, *Cohen's d* = 0.8. Following happy expressions, they seemed to be faster to targets at the location predicted by happy expressions than the other location. However, this difference did not reach statistical significance, *t*_(17)_ = 1.63, *p* = 0.12, *Cohen's d* = 0.4.

In sum, these analyzes suggest a clear learning of the predictive value of expressions type, at least in contingency aware participants. The results suggest a less pronounced learning of the predictive value of expressions in contingency unaware participants.

#### Test phase without context cues

During the test phase without context cues, 2.2% of the responses were excluded (0.3% incorrect responses; 1.9% RT outliers). Mean RTs (Table 1, middle-rows) to targets following morphed expressions were subjected to an ANOVA with target location (2: location predicted by painful expressions vs. location predicted by happy expressions) as within-subjects factor and PCS group (2: high vs. low) as between-subjects factor. This analysis revealed no significant effects, *Fs*_(1, 59)_ < 3.3, *ps* > 0.7, η^2^_*p*_*s* < 0.05. There was no significant correlation between interpretation bias score (i.e., mean RT to targets at the location predicted by happy expressions minus mean RT to targets at the location predicted by painful expressions) and total PCS score, *r*_(61)_ = 0.14, *p* = 0.27.

Adding awareness (2: cue-target contingency-aware vs. unaware) as between-subjects factor to the ANOVA with target location and PCS group as factors revealed a significant 3-way interaction between target location, PCS group, and awareness, *F*_(1, 57)_ = 6.9, *p* = 0.01, η^2^_*p*_ = 0.09. There was no other significant interaction or main effect *Fs* < 1.6, *ps* > 0.2.

For each awareness group separately, mean RTs to targets following morphed expressions were subjected to an ANOVA with target location and PCS group. For participants who were *contingency aware*, there was a significant interaction between target location and PCS group, *F*_(1, 41)_ = 7.9, *p* = 0.007, η^2^_*p*_ = 0.16 [main effects: target location *F*_(1, 41)_ = 2.7, *p* = 0.11, η^2^_*p*_ = 0.06; PCS group *F*_(1, 41)_ < 1]. In line with this finding, in contingency aware participants there was also a significant positive correlation between interpretation bias score and total PCS score, *r*_(43)_ = 0.34, *p* = 0.02, suggesting that higher levels of pain catastrophizing are associated with a more negative interpretation of ambiguous pain-related facial expressions.

**Figure 2:**
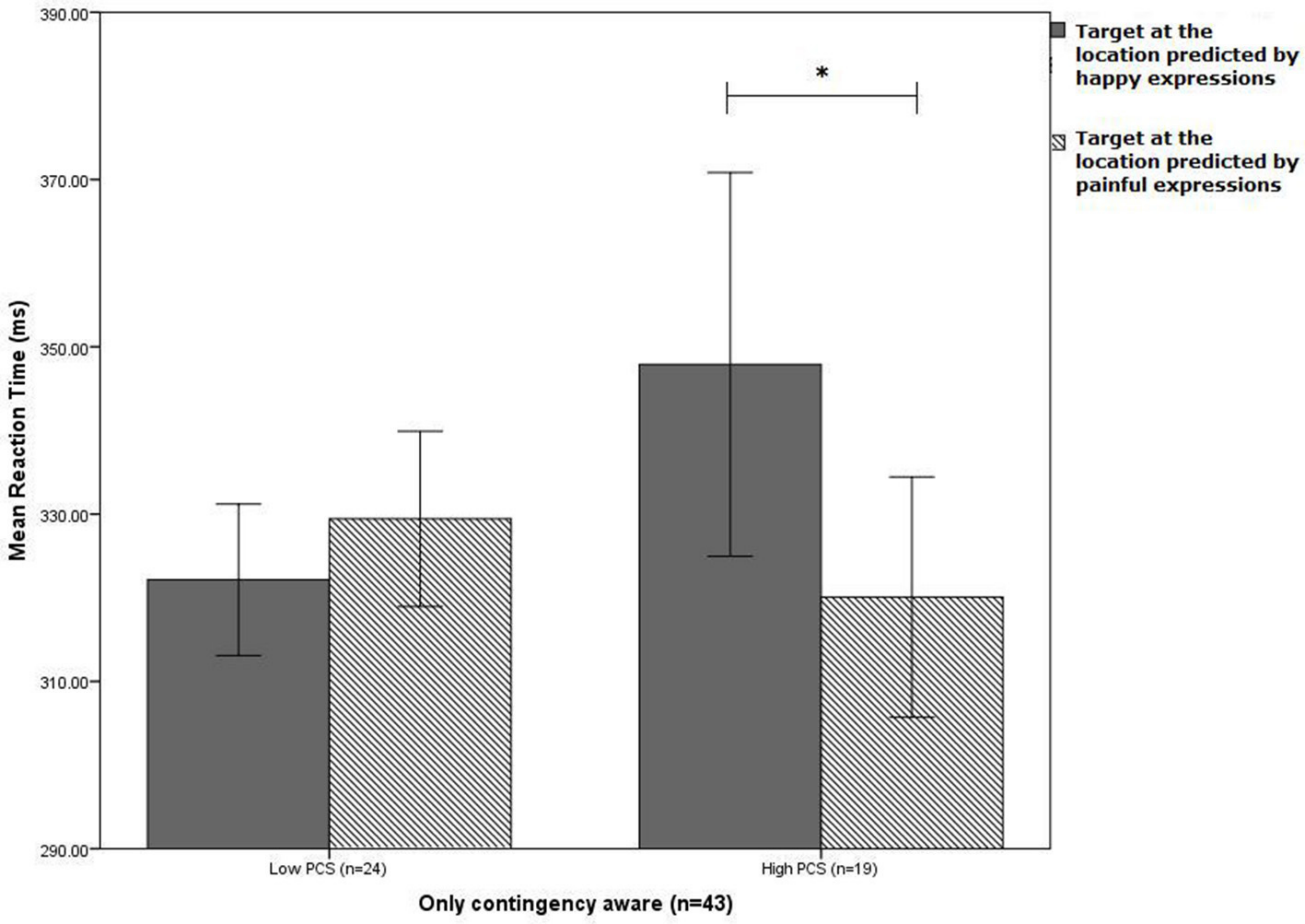
Mean reaction time of participants scoring relatively low and high on the Pain Catastrophizing Scale (PCS) to targets following morphed expressions at the location predicted by painful and happy expressions (^*^p < 0.05, there was no trend or other significant difference) (error bars represent s.e.m.).

As can be seen in Figure 2, among contingency aware participants, high pain-catastrophizers responded faster to targets at the location predicted by painful expressions as compared to targets at the location predicted by happy expressions, *t*_(18)_ = 2.36, *p* = 0.03, *Cohen's d* = 0.34, suggesting biased interpretation toward painful expressions. Low pain-catastrophizers showed no such a difference in their responses *t*_(23)_ = 1.21, *p* = 0.24, *Cohen's d* = 0.15. Figure 2 also suggests that among contingency aware participants, high catastrophizers, as compared to low catastrophizers, were especially slow to targets at the location predicted by happy expressions (*Cohen's d* = 0.35) and that there was no such group difference in responses to targets at the location predicted by painful expressions (*Cohen's d* = 0.17). However, for neither target location, the group difference reached statistical significance [painful faces: *t*_(41)_ = 0.54, *p* = 0.59; happy faces: *t*_(23.6)_ = 1.04, *p* = 0.31, equality of variances not assumed]. For participants who were *unaware* of the contingency, the ANOVA with target location and PCS group revealed no significant effects, *F*_(1,16)_*s* < 2.6, *ps* > 0.13, η^2^_*p*_*s* > 0.14. This group showed also no significant correlation between interpretation bias score and PCS score, *r*_(18)_ = 0.2, *p* = 0.15.

#### Test phase with context cues

During the test phase with context cues, 2.8% of the responses were excluded from analysis (0.3% incorrect responses; 2.5% RT outliers). Mean RTs (Table 1, lower rows) to targets following morphed expressions were subjected to an ANOVA with target-location (2: location predicted by happy expressions vs. location predicted by painful expressions) and context cue (2: threatening vs. non-threatening) as within-subjects factors and PCS group (2: high vs. low) as between-subjects factor. Overall, responses were slower following non-threatening cues than following threatening cues, *F*_(1, 59)_ = 6.0, *p* = 0.017, η^2^_*p*_ = 0.09. There were no other significant effects: *Fs*_(1, 59)_ < 2.5, *ps* > 0.1, η^2^_*p*_*s* < 0.04. Including awareness as additional factor revealed no further significant effects.

### Direct classification task

The prototype happy and painful faces were 100% correctly categorized. The number of morphs classified as painful or happy were subjected to an ANOVA with classification (2: classified as painful vs. happy) as within-subject factor and PCS group (2: high vs. low) as between-subjects factors. This analysis revealed a significant main effect of classification, *F*_(1, 59)_ = 41.5, *p* < 0.001, η^2^_*p*_ = 0.4, but no effect of PCS group [main effect PCS group: *F*_(1, 59)_ < 1; PCS group × classification: *F*_(1, 59)_ < 1]. As can be seen in Table 2, morphs were categorized nearly twice as often as happy than as painful, irrespective of pain catastrophizing level. There was no significant correlation between the percentage of morphed expressions classified as painful (vs. happy) and total PCS score, *r*_(61)_ = 0.1, *p* = 0.5. There was also no significant correlation between interpretation bias scores on the incidental learning task and percentage of morphed expressions classified as painful on the direct classification task, neither overall *r*_(61)_ = −0.04, *p* = 0.8, nor per PCS group or contingency awareness group *rs* < 0.1, *ps* > 0.6.

**Table 2:** **Classifications of the 16 morphed expressions during the direct classification task separately for those scoring low and high on the Pain Catastrophizing Scale (PCS)**.

	**Low PCS**	**High PCS**
	***n* = 32**	***n* = 29**
Mean number of expressions classified as painful (±*SD*)	5.5 ± 2.9	5.4 ± 3.1
Mean number of expressions classified as happy (±S*D*)	10.2 ± 2.8	10.4 ± 3.2

## Discussion

The primary objective of this study was to investigate whether healthy individuals, especially those with higher levels of pain catastrophizing, show a negative interpretation bias for ambiguous pain-related facial expressions. Secondly, the effect of threatening contextual information on individuals' interpretation of ambiguous expressions was evaluated. Interpretation bias was assessed using an indirect as well as a direct measure.

The results can be summarized as follows. *First*, following morphed expressions during the incidental learning task, and only among contingency-aware subjects, individuals with relatively high levels of pain catastrophizing responded faster to targets appearing at the location predicted by painful expressions than to targets at the location predicted by happy expressions, while this was not the case for the low pain catastrophizers. High pain catastrophizers were also slower in reacting to targets at the location predicted by happy expressions and slightly faster to targets at the location predicted by painful expressions, although neither of these two differences reached standard levels of statistical significance. *Second*, when contextual cues were included in the incidental learning task, there was no indication of interpretation bias. Overall responses were slower following presentation of non-threatening contextual cues than threatening contextual cues. *Third*, independent of their level of catastrophizing, participants classified the morphed facial expressions more often as happy than as painful.

The response pattern as shown by high catastrophizers during the incidental learning task can be taken to reflect a threat-related interpretation bias toward pain. This finding is in line with previous research showing that negative interpretation of bodily sensations is associated with higher levels of catastrophizing in healthy individuals (Vancleef and Peters, 2008). It is suggested that negative interpretation bias plays a role in the development of pain-related problems as seen in individuals with high levels of pain-related catastrophizing (Pincus and Morley, 2001).

It is noteworthy that in our study, the interpretation bias effect was observed only among participants with relatively high levels of pain catastrophizing who also reported awareness of the association between expression type and target location. Modern accounts of associative learning (Mitchell et al., 2009) and evaluative conditioning (Kattner, 2012), assume that contingency awareness is a prerequisite for learning to occur, and our findings are in line with this assumption. However, further studies are needed to further evaluate the effect and importance of contingency-awareness during incidental learning paradigms.

Our study extends previous research in at least two ways. *First*, it shows catastrophizing-related differences in interpretation of morphed painful expressions. Previous studies on pain-related interpretation bias primarily focused on the biased processing of ambiguous words related to pain and somatic threat (Edwards and Pearce, 1994; Pincus et al., 1994). The few studies on biased interpretation of ambiguous pain-related expressions (Yamada and Decety, 2009; Liossi et al., 2012) did not take into account the individual differences in the interpretation of ambiguous expressions in a pain-free population, and used only direct measures of interpretation.

*Second*, this study is to our knowledge the first in applying both an indirect and a direct measure to examine pain-related interpretation bias for the same stimulus material, in the same sample, and during the same session. Interestingly, those indirect and direct measures seemed to reveal different outcomes. During the direct classification task, participants classified morphed expressions more often as happy, suggesting a biased interpretation toward happy expressions independent of pain catastrophizing. This finding is in line with some previous observations, for example of mothers directly classifying ambiguous painful-happy expressions more often as happy than as painful (Liossi et al., 2012). In the incidental learning task, interpretation bias depended on subjects' level of catastrophizing (more negative interpretation of ambiguity among high catastrophizers and neutral interpretation among low catastrophizers). Structural differences between the direct classification task and the indirect incidental learning task might help to explain differences in results. Differences might for example be due to the participant's level of control on the outcome of the to-be-measured bias. The outcome of direct measures are directly based on participants' response, while in the indirect measures the responses will be derived from performance behavior (De Houwer and Moors, 2010). In experiments with direct measures it is easier for participants to be aware of the goal of research, as compared to those with indirect measures. Being aware of crucial stimuli during direct measures does more likely change the subjects' attribute which might influence the performance during the task. Further research is needed to systematically study structural differences between direct and indirect measures of interpretation bias, the precise mechanisms that underlie them, and the characteristics of the interpretation biases that are captured.

When contextual cues preceded the expressions in the incidental learning task, there was no indication of expression-related and/or catastrophizing-related differences between responses to targets following morphed expressions at all. This observation corroborates to a certain extent a previous finding showing that healthy individuals' sensitivity to the presence of pain in ambiguous facial expressions is independent from the affective value of a prime (Yamada and Decety, 2009). However, the results of this previous study also showed that, in contrast to the present findings, the tendency to actually classify ambiguous pain-related facial expressions as painful is especially enhanced when these expressions are preceded by negative priming words. One possible but speculative explanation is that in the current study incidental learning effects were overridden by masking/priming effects by the contextual cues. Note that including contextual cues resulted in overall slower reaction times to targets and increased variance (Table 1). Finally, perceived direction of threat may have an influence on the priming of expressions by contextual cues. This is an interesting avenue for future studies to consider the effect of different pain reference frames (self vs. other) between contextual cues and ambiguous pain stimuli on interpretation bias.

A number of limitations should be acknowledged when interpreting the current findings. *First*, the present sample was female. Previous studies showed that there is a relationship between negative interpretation bias and measures of pain-related anxiety only among females and not males (Keogh et al., 2004). In this first study on catastrophizing-related interpretation bias for ambiguous pain-related facial expressions, with the incidental learning task, we wanted to avoid any influence of gender and therefore chose for a female-only sample. However, we also recognize that using a relatively homogeneous, female-only student sample limits the generalizability of results. It would therefore be valuable for further research to also consider samples of balanced gender and different age groups to strengthen the external validity of the findings. Replication of a similar experimental approach in a clinical sample would help us to understand the role of interpretation bias in chronic pain and dysfunctional pain behavior. *Second*, to test the main hypothesis of this study we only included painful-happy morphs. In order to study the content-specificity of the observed effects, other emotionally ambiguous expressions, such as morphs between happy expressions and expressions of negative emotions (e.g., anger, sadness) might be included. *Third*, pictorial face stimuli were carefully chosen and created based on ratings as delivered with the original databases, ratings by an independent group of participants drawn from the same population as the current sample, and ratings by experts in facial coding. The created morphs were also rated as ambiguous by the participants of the actual experiment (see Section Pictorial Face Stimuli). Future studies might prefer to use stimuli that are selected based on ratings in a bigger and more diverse sample, also taking individual differences among raters into account. It would also be valuable for future studies to have participants themselves rate intensity of emotions in the facial expressions and to also take into account other facial cues (e.g., age, race, sex). As an alternative approach, in order to avoid pre-selection of ambiguous stimuli based on subjective ratings, one might present morphs with different intensities of expressions and use a signal detection approach (as in Liossi et al., 2012) or derive psychophysical functions to examine the relationship between negative interpretation bias in ambiguous pain-related expressions and pain catastrophizing.

Taken together, to our knowledge this study is the first study that used an incidental learning task (in addition to a direct classification task) to investigate pain-related interpretation bias, and more specifically interpretation bias for ambiguous facial expressions in catastrophizing. The observed biased interpretation of ambiguous pain-related expressions is relevant in the context of observational learning and its presumed role in the development of pain problems. It has for example been suggested that a pain-related interpretation of ambiguous pain signals, as for example expressed by the behavior of others, is associated with the acquisition of pain-related fear in response to that painful expression (Goubert et al., 2011). Recent research shows that pain-free participants who observe others immersing their hand in assumed cold water, before performing the same immersion task themselves, express more pain-related fear and expect more unpleasant and intense pain when the color of the water is associated with painful rather than with neutral facial expressions (Helsen et al., 2012). This acquisition process is likely to be mediated by the interpretation of the model's expression. Further research is warranted to test these presumed causal mechanisms systematically.

### Conflict of interest statement

The authors declare that the research was conducted in the absence of any commercial or financial relationships that could be construed as a potential conflict of interest.

## Footnotes

^1^http://www.fantamorph.com

^2^Contingency aware and unaware participants did not significantly differ in pain catastrophizing, as suggested by a univariate ANOVA with PCS group (2: high vs. low PCS) and awareness (2: cue-target contingency-aware vs. contingency-unaware) as between-group factors and PCS total score as dependent variable [main effect awareness: *F* < 1; PCS group × awareness: *F* < 1; main effect of PCS group *F*(1, 57) = 89.2, *p* < 0.001, η^2^_*p*_ = 0.61].
