# Supplementary material for: An experimental examination of catastrophizing-related interpretation bias for ambiguous facial expressions of pain using an incidental learning task
Source: Front Psychol. 2014 Sep 17;5:1002. doi: 10.3389/fpsyg.2014.01002 (PMC4166218; doi:10.3389/fpsyg.2014.01002)
Supplement: Supplementary file 1 [file Table1.DOCX]

| **Table S1.**  *Rating (mean ± sd) of Happy and Painful Expressions on Four Likert Scales (Intensity of Happiness, Intensity of Pain, Perceived Pleasantness, and Arousal) Separately for Those Included as Non-Ambiguous (unmorphed) Expressions and Those Used for Creating Ambiguous Expressions (morphs).* | | | | | | |
| --- | --- | --- | --- | --- | --- | --- |
|  | unmorphed stimuli | | | morphed stimuli | | |
|  | Happy expression | Painful expression | *t(19)* | Happy expression | Painful expression | *t(19)* |
| Happiness  [0 to 5 scale] | 3.22 ± 0.44 | 0.25 ± 0.22 | 27.00 ^*^ | 3.43 ± 0.33 | 0.21 ± 0.19 | 37.81 ^*^ |
| Pain  [0 to 5 scale] | 0.24 ± 0.18 | 3.75 ± 0.57 | 26.26 ^*^ | 0.27 ± 0.19 | 3.78 ± 0.43 | 33.39 ^*^ |
| Pleasantness  [-4 to 4 scale] | 2.79 ± 0.59 | -3.06 ± 0.46 | 34.97 ^*^ | 2.69 ± 0.62 | -2.87 ± 0.51 | 30.97 ^*^ |
| Arousal  [-4 to 4 scale] | -2.34 ± 0.27 | 2.58 ± 0.51 | 38.13 ^*^ | -2.44 ± 0.31 | 2.61 ± 0.62 | 32.58 ^*^ |
|  | | | | | | |
| ^*^ *p < 0.001*  *statistics for pairwise comparison for painful and happy expressions is given.* | | | | | | |
