# Supplementary figures and images for: An experimental examination of catastrophizing-related interpretation bias for ambiguous facial expressions of pain using an incidental learning task
Source: Front Psychol. 2014 Sep 17;5:1002. doi: 10.3389/fpsyg.2014.01002 (PMC4166218; doi:10.3389/fpsyg.2014.01002)

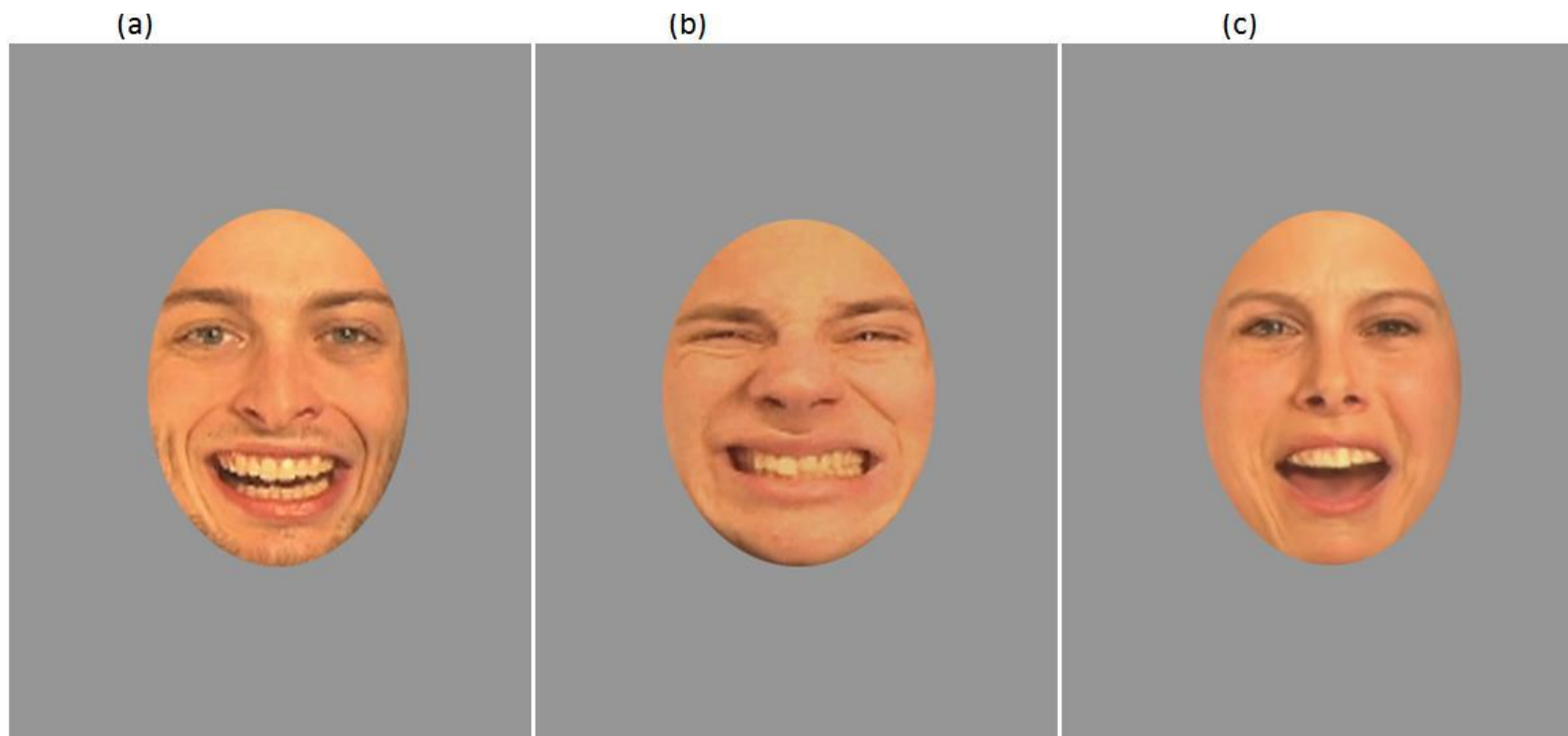

**Figure S1.** Examples of (a) happy, (b) painful, (c) morphed expressions used in our study

Supplement: Supplementary file 2 [file Image1.PDF]

(a)

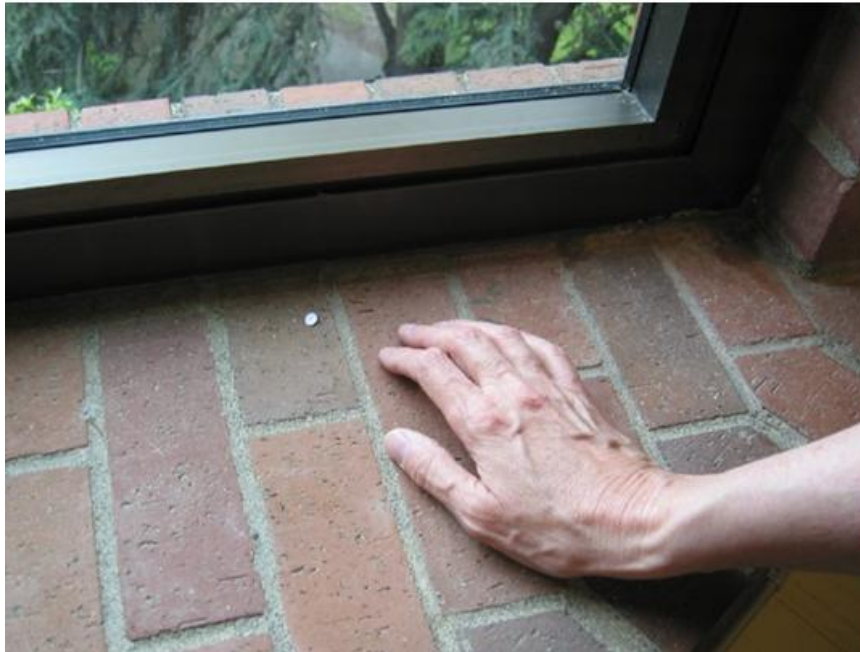

(b)

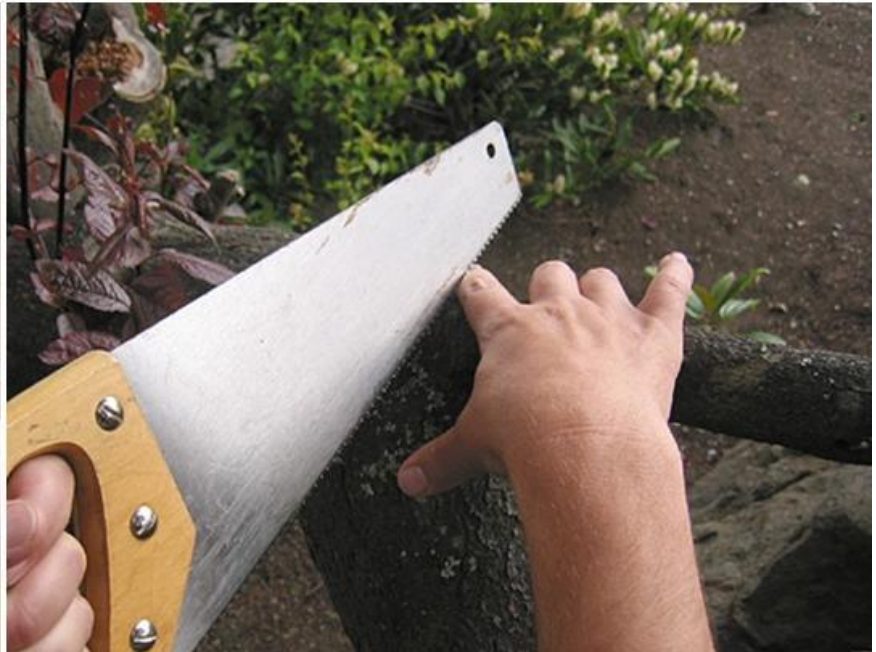

**Figure S2.** Examples of contextual cues used in our study (a) Neutral (b) Painful cues

Supplement: Supplementary file 3 [file Image2.PDF]
